# Molecular Testing in Organ Biopsies and Perfusion Fluid Samples from Severe Acute Respiratory Syndrome Coronavirus 2 Positive Donors

**DOI:** 10.3390/v17121611

**Published:** 2025-12-13

**Authors:** Evangelia Petrisli, Liliana Gabrielli, Carlo De Cillia, Andrea Liberatore, Giulia Piccirilli, Simona Venturoli, Alice Balboni, Eva Caterina Borgatti, Alessia Cantiani, Lamberto Manzoli, Nicola Alvaro, Tiziana Lazzarotto

**Affiliations:** 1 Microbiology Unit, IRCCS Azienda Ospedaliero-Universitaria di Bologna, 40138 Bologna, Italy; 2 Emilia Romagna Transplant Reference Centre, IRCCS Azienda Ospedaliero-Universitaria di Bologna, 40138 Bologna, Italy; 3 Department of Medical and Surgical Sciences, Section of Microbiology, University of Bologna, 40138 Bologna, Italy; 4 Department of Environmental and Prevention Sciences, University of Ferrara, 44121 Ferrara, Italy

**Keywords:** SARS-CoV-2, donor-derived infection, transmission, graft biopsy, organ perfusion fluid

## Abstract

At the beginning of the COVID-19 pandemic, SARS-CoV-2-positive donors were not considered eligible for organ donation. The Italian National Transplant Centre has gradually introduced measures to prevent donor-to-recipient transmission of SARS-CoV-2 infection through organ transplantation. The current national screening protocol for deceased SARS-CoV-2-positive donors recommends molecular testing of donor lower respiratory tract (LRT) samples, graft biopsies and organ perfusion fluids. The aim of the study is to describe the 3-year experience of protocol application in a northern region of Italy. From 1 January 2022 to 31 January 2025, a total of 132 samples were analyzed (29 liver biopsies, 35 kidney biopsies, 68 perfusion fluids) from 40 organ donors with an active or resolved SARS-CoV-2 infection. SARS-CoV-2 PCR on LRT samples was positive in 26/40 (65%) donors, negative in 11/40 (27.5%) cases and in the remaining 3 (7.5%) the PCR result was unknown. Overall, 65 organs were transplanted into 60 recipients. All processed graft biopsies and organ perfusion fluid samples tested negative for SARS-CoV-2 RNA. Our data suggest that the utilization of non-lung donors with resolved or active SARS-CoV-2 infections who died of other causes appears justified and safe.

## 1. Introduction

On 11 March 2020, the World Health Organization declared the coronavirus disease 2019 (COVID-19) outbreak a global pandemic [1]. This represented an unprecedented event in recent human history with a major impact on the organ donation and transplantation fields due to the threat it represented in terms of safety, sufficiency and access to these lifesaving procedures. Uncertainties regarding RNAemia or the viral presence in bodily fluids during the incubation period, during an asymptomatic course of infection, or after symptom resolution posed the risk of severe acute respiratory syndrome coronavirus 2 (SARS-CoV-2) transmission from donor to recipient in a period of high community infection rates worldwide. Evidence of postmortem detection of SARS-CoV-2 in the heart, kidney, lungs, liver, spleen, colon, brain and bone marrow as well as inflammatory damage to the graft has also raised concern [2]. As a precautionary action to mitigate the potential infectious risk during the initial wave of the pandemic, international transplantation societies and national competent authorities recommended living- and deceased-donor SARS-CoV-2 screening by molecular testing on respiratory specimens before organ procurement and, in cases of positive results, donor deferral in order to prevent unintended harm to the recipients [3].

The first case of proven donor-transmitted SARS-CoV-2 infection to a bilateral lung recipient and to a thoracic surgeon involved in the preparation and implantation of the lungs was published in 2020 [4]. The donor nasopharyngeal swab (NPS) SARS-CoV-2 PCR prior to procurement was negative but retrospectively tested positive in a lower respiratory tract specimen. This led to the recommendation to use lower respiratory tract (LRT) sample testing during deceased donor characterization, particularly in the case of lung donation [5]. Knowledge significantly increased over time through the emerging literature and led to the initiation of SARS-CoV-2-positive allograft utilization protocols in order maintain or expand the donor pool without compromising safety. The Italian National Transplant Centre (CNT) implemented protocols for transplantation from donors with resolved infection of at least 14 days in May 2020 (this interval has been reduced to 7 days starting from January 2022 [6]). Subsequently, a protocol to use organs from highly selected donors with active SARS-CoV-2 infection was introduced in November 2020 for liver and heart recipients and in January 2022 for kidney recipients [7].

The current national screening protocol for deceased SARS-CoV-2-positive organ donors recommends molecular testing for SARS-CoV-2 on donor LRT samples as well as on biopsy and organ perfusion fluid samples, whereas biopsy of the heart graft is left at the discretion of the transplant center [8]. This study reports on the virological results obtained by molecular testing on graft biopsies and perfusion fluid samples of organs procured from deceased donors with resolved or active SARS-CoV-2 infection processed at the Microbiology Unit of the IRCCS St. Orsola Polyclinic, Bologna, Italy.

## 2. Materials and Methods

Donors were determined eligible for donation according to the Italian regulatory requirements. Samples were analyzed in the Laboratory of Virology and the Regional Reference Center for Microbiological Emergencies CRREM-Microbiology Unit of the IRCCS St. Orsola Polyclinic, Bologna, Italy. Donors underwent SARS-CoV-2 RT-PCR in bronchoaspirate (BAS) or bronchoalveolar lavage (BAL) samples, liver or kidney intra-operative biopsy and graft perfusion fluid samples at organ recovery. Graft biopsies and preservation fluid samples of SARS-CoV-2-negative donors were not available for testing. All recipients were tested for SARS-CoV-2 nucleic acid in NPS immediately before transplant. Furthermore, informed consent was signed at the time of the organ candidate’s registration in the transplant waiting list or before transplant, and the determination of IgG anti-SARS-CoV-2 titer was also foreseen.

Results were communicated to the Emilia Romagna Transplant Reference Centre within 48 h after transplant. According to Italian law, Regional Transplant Centers are the custodians of donor/recipient biomedical data also for research purposes [9]. All study procedures complied with the ethical standards of the 2000 Declaration of Helsinki and the 2008 Declaration of Istanbul.

SARS-CoV-2 PCR in BAL or BAS was performed using Xpert^®^ Xpress SARS-CoV-2 on the GeneXpert instrument (Cepheid Italia, Milano, Italy), a qualitative assay for the detection of SARS-CoV-2-specific genomic regions coding for the nucleocapsid (N2) and envelope (E) protein. The analytical limit of detection (LoD, i.e., lowest concentration of live SARS-CoV-2 virus samples that can be reproducibly distinguished from negative samples ≥ 95% of the time with 95% confidence, reported as PFU/mL) is 0.0200 PFU/mL.

Qualitative and quantitative multiplex nucleic acid reverse transcription and amplification for the detection of the genomic RNA of SARS-CoV-2 in graft biopsies and perfusion fluid samples was performed using the ELITeInGenius SP 200 and SARS-CoV-2 ELITe MGB^®^ Kits (ELITechGroup, Torino, Italy) on the ELITeInGenius^®^ (ELITechGroup) instrument. Specifically for biopsies, a sample sized between 3 and 4 mm in diameter was placed in a 2 mL tube with 270 μL of tissue lysis buffer and 30 μL of protease (ATL buffer and Proteinase K solution, respectively; Qiagen Italia, Milano, Italy). After overnight incubation at 56 °C, nucleic acids were extracted according to manufacturer instructions.

The assay detects the RNA of two specific genomic regions: RNA-dependent RNA polymerase (RdRp) and ORF8 genes. The complete reaction mixture also amplifies the cellularity, extraction and inhibition control based on the human RNase P gene as endogenous internal control, allowing confirmation of the quality of the sample material extracted. The limit of detection of the test on biopsy and perfusion fluid samples is 2 copies/reaction and 100 copies/mL, respectively.

Humoral response to SARS-CoV-2 infection or vaccination has been determined by the Elecsys^®^ Anti-SARS-CoV-2S immunoassay (Roche Diagnostics AG, Rotkreuz, Switzerland) performed on the cobas e 801 analyzer (Roche Diagnostics). The test is a quantitative electrochemiluminescence immunoassay that detects antibodies to the SARS-CoV-2 S protein receptor-binding domain (RBD), which binds to the angiotensin-converting enzyme-2 (ACE2) receptor and mediates the initial step of virus fusion with the host cell and correlates well with surrogate neutralization assays [10]. Results are reported as the analyte concentration of each sample in U/mL. The cut-off value for positivity of RBD-specific antibodies is 0.8 U/mL, whereas the upper instrumental limit of detection is 2500 U/mL.

## 3. Results

**Figure 1:**
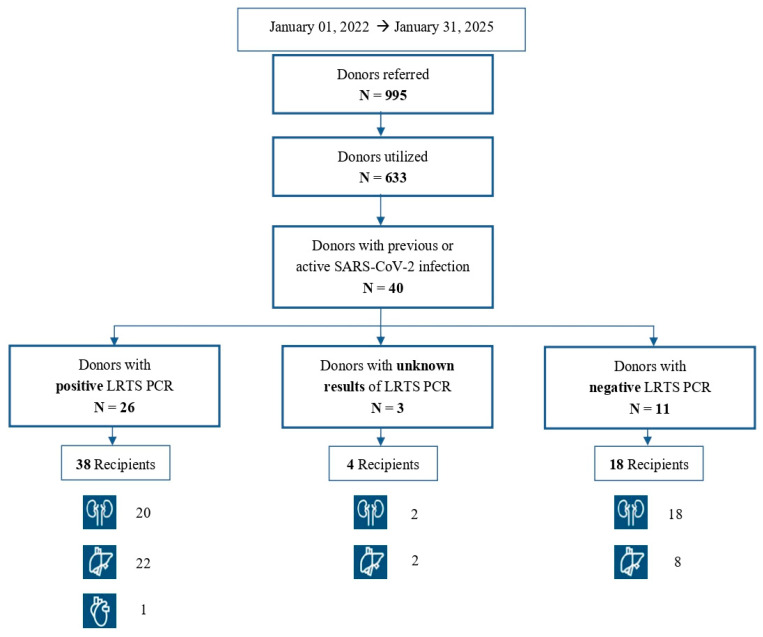
Summary of the overall donors, associated recipients and organs procured during the study period.

From 1 January 2022 to 31 January 2025, 995 potential organ donors were referred to the Emilia Romagna Transplant Reference Centre, but ultimately only 633 donors were utilized (214 oppositions to donation; 148 donors unsuitable for donation). Among them, 40 (40/633; 6%) were organ donors with an active or resolved SARS-CoV-2 infection and donated organs to a total of 60 recipients. The summary of the overall donors, associated recipients and organs procured is outlined in Figure 1.

### 3.1. Donors

Organs were procured from donors after meeting brain or circulatory death criteria. The median age of the donors was 63 years. Twenty-six of the subjects (65%) had an active SARS-CoV-2 infection with viral RNA detection in BAS or BAL samples tested at organ procurement. Among SARS-CoV-2-positive donors, the cycle threshold (Ct) value was known in 22 of the cases (average Ct 29.5; range: 14–41) and 12/26 of them (46%) had LRT samples with Ct levels lower than 30 (range 14–29). In 3/40 (7.5%) cases, the respiratory sample PCR result was not reported (one extra European Union donor; two extra Emilia Romagna region donors). Further details of donor characteristics are outlined in Table 1.

All donors were asymptomatic, whereas anti-SARS-CoV-2 serostatus was available for only four donors that resulted IgG-positive at the time of organ recovery. All donors died of causes other than COVID-19. 

By protocol, organs underwent biopsy at procurement for histological evaluation and SARS-CoV-2 molecular testing on graft biopsies and perfusion fluids. A total of 132 samples were processed: 29 liver biopsies, 35 kidney biopsies and 68 preservation fluids. The heart biopsy was not performed.

SARS-CoV-2 RNA PCR was negative in 100% of the samples tested.

### 3.2. Recipients

A total of 73 organs were procured; 65 organs were suitable for transplantation, whereas 8 were declined by the transplant center. Overall, 32 livers, 32 kidneys and 1 heart were donated to 60 recipients. The median recipient age was 57 years (range 21–74), and the male/female ratio was 47/13. Indications for transplant and further details of recipient characteristics are outlined in Table 2.

All recipients underwent NPS PCR pre-operatively on post-operative day 7 and subsequently on a weekly basis for one month post-transplant. The totality of respiratory samples processed resulted PCR-negative at procurement and during follow-up.

Among the 60 recipients, 23 (38%) had received SARS-CoV-2 vaccination prior to transplant. Overall, a total of 33 recipients had a known anti-SARS-CoV-2 serostatus at the time of organ recovery and resulted anti-SARS-CoV-2 IgG-positive in 32 cases (32/60; 53%) and negative in 1 case. The anti-RBD-specific IgG titer was higher than the upper limit of assay detection (>2500 U/mL) in 29/32 seropositive recipients.

## 4. Discussion

The beginning of the COVID-19 pandemic has registered a dramatic impact on the transplantation field globally with a significant reduction in transplant rates [11]. Experience significantly increased over time through scientific evidence and the initiation of SARS-CoV-2-positive allograft utilization protocols were gradually introduced. Based on the current national screening protocol, we present the virological data obtained by molecular testing on graft biopsies and perfusion fluid samples of organs procured from deceased donors with resolved or active SARS-CoV-2 infection to non-lung solid organ recipients of the Emilia Romagna region during a 3-year period. To our knowledge, this is the largest study reporting on 64 graft biopsies and 68 perfusion fluid samples from 40 deceased donors with resolved or active SARS-CoV-2 infection.

In our cohort, 26/40 (65%) of the donors were experiencing an active SARS-CoV-2 infection at the time of organ procurement and almost half of them (12/26; 46%) had LRT samples with Ct levels lower than 30, implying a high viral load in the specimens. Nevertheless, no donor-to-recipient SARS-CoV-2 transmission was observed during the study period. Of note, no additional testing to discriminate between active viral replication and free viral RNA fragments was performed in our study. Ct value-based approaches have been used to presume infectivity and distinguish between active viral replication and prolonged virus shedding [12,13]. However, definitive data to support the predictive value of Ct values in these situations are lacking due to numerous factors that are inherent to the qualitative PCR assays and respiratory specimen characteristics. In fact, Ct values in qualitative PCR assays are not normalized to standardized controls of known concentrations and are also not optimized to have a linear relationship between Ct value and concentration of target nucleic acid in the specimen as compared to the quantitative PCR assays. Furthermore, unlike, for example, blood samples, the standardization of respiratory specimens is more challenging in terms of adequacy of specimen collection, dilution in transport medium and storage conditions [14]. To overcome these limitations, some studies have suggested that subgenomic RNAs (sgRNAs) can be used as surrogates of SARS-CoV-2 infectivity since their formation occurs only during active replication. Santos Bravo et al. assessed sgRNA as a surrogate marker of viral infectivity in 105 SARS-CoV-2-positive RT-PCR respiratory samples in comparison with the gold standard for virus replication represented by viral culture. The authors reported that sgRNA and viral isolation results were concordant in 99/105 cases (94%), indicating highly significant agreement between the two techniques [15]. Saharia et al. described a successful lung transplantation where the donor lung tissue was retrospectively found to have detectable SARS-CoV-2 RNA by multiple assays including highly sensitive droplet digital PCR, viral sequencing and in situ hybridization; however, sgRNA was not present despite the detection of genomic viral RNA in the donor tissue, suggesting the absence of replicating virus [16]. Further studies are needed to assess the use of subgenomic RNA as a surrogate marker of active virus replication, which could help to mitigate the risk of infection transmission, enhance organ utilization and expand the donor pool.

Although there are numerous reports of SARS-CoV-2 detection in different organs, tissues and cells, including autopsy studies, data regarding graft-specific infection status in SARS-CoV-2-positive donors have been rarely described. In our study population, molecular testing for SARS-CoV-2 RNA on 64 graft biopsies and 68 perfusion fluid samples at procurement was negative in 100% of cases, including donors with LRT samples with Ct levels lower than 30, suggesting a very low risk of donor-transmitted infection with kidney or liver transplantation. Romagnoli et al. reported negative SARS-CoV-2 RNA PCR results obtained from liver biopsies in nine of nine tested active COVID-19 donors, suggesting a very low risk of transmission with liver transplantation [9]. Martini et al. [17] reported no donor-derived transmission among 25 recipients receiving a liver transplant from 25 donors with active COVID-19 (n = 24) or history of COVID-19 2 months before death (n = 1). Among the 21 donors who underwent SARS-CoV-2 PCR in liver biopsy, 20 resulted negative and 1 positive (the corresponding recipient underwent re-transplant after 107 days due to hepatic artery thrombosis). In a two-center study of 26 kidney transplants performed from 24 SARS-CoV-2 PCR-positive deceased donors, the authors reported the absence of detectable viral RNA in 24 donor plasma/serum samples and 26 pre-transplant kidney biopsy samples, including those from 10 donors with symptomatic disease (SARS-CoV-2 RT-PCR Ct was available only for 12 donors, range: 28–40) [13]. Jashari et al. described two SARS-CoV-2-positive recipients of heart transplantation who donated their failed hearts in a domino procedure for valve transplantation [18]. In both cases, myocardial tissue, transport solution used for shipment and sample sets of the mitral and tricuspid leaflets of both hearts were SARS-CoV-2 PCR-negative. Finally, Whitrock et al. reported no donor-derived transmission events among 20 recipients undergoing liver transplantation from SARS-CoV-2-positive deceased donors, with only 2 (2/20; 10%) post-reperfusion allograft biopsies positive for viral RNA detected by droplet digital polymerase chain reaction testing [19]. Nevertheless, the interpretation of results from such ultrasensitive detection methods must be made with caution and the distinction between presence of genomic material and its ability to infect and replicate in vivo should be further investigated.

Over time, the continuous viral circulation resulting in high community infection rates, as well as the introduction of effective vaccines, led to a significant rise in immunity against SARS-CoV-2 in the general population. SARS-CoV-2 vaccines have been rapidly developed and mass vaccination programs began in December 2020. Most countries have adopted vaccination prioritization processes, including priority for solid organ transplant recipients and candidates on the waitlist [20]. A survey regarding the current transplantation practices in Member States of the Council of Europe reported that most waitlisted patients receiving organs from a deceased donor with active or resolved SARS-CoV-2 infection were preferred to have natural, vaccine-induced or hybrid SARS-CoV-2 immunity [21]. In our study population, IgG against SARS-CoV-2 were positive in 32/33 recipients with a known anti-SARS-CoV-2 serostatus at transplant, with the majority (29/32) having an anti-RBD-specific IgG titer higher than the upper limit of assay detection (>2500 U/mL), although it has not been possible to differentiate between natural, vaccine-induced or hybrid SARS-CoV-2 immunity.

In May 2023, due to a significant improvement in the global epidemiological situation and reinforced by the growing scientific knowledge in terms of recipient outcomes, accurate diagnostics, vaccination and available treatment options, the World Health Organization declared that COVID-19 no longer constitutes a public health emergency of international concern [22]. Successively, the European Centre for Disease Prevention and Control issued the last update on the safety of substances of human origin in relation to COVID-19 and assessed the incidence of donor-to-recipient transmission of SARS-CoV-2 as negligible, stating that, except for the lungs and potentially intestine transplantation, there is no evidence of a risk [23].

Our findings are subjected to some limitations, including the small sample size and lack of information that could potentially impact the post-transplant clinical course, such as granular data regarding previous donor and recipient SARS-CoV-2 immunity, administration of post-transplant antiviral therapy to the recipients and short- and long-term transplant-related outcomes.

In conclusion, in accordance with the current scientific evidence, our data provides additional evidence that the utilization of non-lung organs from deceased donors with active or resolved SARS-CoV-2 infection to informed transplant candidates appears justified and safe.

## Figures and Tables

**Table 1:** Characteristics of donors with active or resolved SARS-CoV-2 infection.

DONORS (N = 40)	
Median age, years (min–max)	63 (20–85)
Male sex [n (%)]	27 (67.5%)
**Type of donor [n (%)]**	
DBD	32 (80%)
DCD	8 (20%)
**Cause of death [n (%)]**	
Cerebral hemorrhage	21 (52.5%)
Stroke/Post-anoxicencephalopathy	11 (27.5%)
Polytrauma/Head Trauma	7 (17.5%)
Hypoglycemic coma	1 (2.5%)
**LRTS SARS-CoV-2 PCR at organ procurement [n (%)]**	
Positive	26 (65%)
Negative	11 (27.5%)
Not available	3 (7.5%)
**Cycle threshold (Ct)**	
>30	10
<30	12
Not available	4

Abbreviations: DBD: donation after brain death; DCD: donation after cardiac death; LRTS: lower respiratory tract sample; SARS-CoV-2: severe acute respiratory syndrome coronavirus 2; PCR: polymerase chain reaction.

**Table 2:** Characteristics of organ recipients receiving graft from donors with active or resolved SARS-CoV-2 infection.

RECIPIENTS (N = 60)	
Median age, years (min–max)	57 (21–74)
Male sex [n (%)]	47 (78%)
**Underlying liver disease**	
Hepatocellular carcinoma	13
Laënnec’s cirrhosis	7
Cryptogenic cirrhosis	3
Biliary cirrhosis	1
Drug-induced cirrhosis	1
Posthepatitic cirrhosis	2
Polycystic liver disease	1
Dysmetabolic liver disease	1
Previous graft rejection	1
**Underlying kidney disease**	
Chronic glomerulonephritis	4
IgA nephropathy	4
Diabetic nephropathy	4
Chronic renal failure	4
Renal hypoplasia	3
Lupus nephritis	2
Polycystic kidney disease	3
Hypertensive nephrosclerosis	1
Focal segmental glomerulosclerosis	1
Renal carcinoma	1
Chronic pyelonephritis	1
**Underlying heart disease**	
Hypertrophic cardiomyopathy	1
**Transplant type [n (%)]**	
Liver	32 (49%)
Kidney	32 (49%)
Heart	1 (2%)
**Recipient serostatus at the time of transplant [n (%)]**	
IgG+	32 (53%)
IgG−	1 (2%)
Not known	27 (45%)

## Data Availability

The original contributions presented in this study are included in the article. Further inquiries can be directed to the corresponding author.

## Abbreviations

The following abbreviations are used in this manuscript:

ACE2	angiotensin-converting enzyme-2
BAL	bronchoalveolar lavage
BAS	bronchoaspirate
CNT	National Transplant Centre (of Italy)
COVID-19	coronavirus disease 2019
Ct	cycle threshold
DBD	donation after brain death
DCD	donation after cardiac death
LRT	lower respiratory tract
NPS	nasopharyngeal swab
PCR	polymerase chain reaction
RBD	receptor-binding domain
RdRp	RNA-dependent RNA polymerase
SARS-CoV-2	severe acute respiratory syndrome coronavirus 2
sgRNAs	subgenomic RNAs
